# Gut Microbiome Dysbiosis Promotes Gallstone Formation via Bile Acid Metabolic Disorder: A Multiomics Study

**DOI:** 10.1096/fj.202503254RRRRRR

**Published:** 2026-03-09

**Authors:** Chongfei Huang, Weijun Xiao, Jinyu Zhao, Ruyang Zhong, Long Gao, Haidong Ma, Liang Tian, Ping Yue, Yanyan Lin, Qiangsheng He, Bin Xia, Jinqiu Yuan, Man Yang, Wenbo Meng

**Affiliations:** ^1^ The First School of Clinical Medicine Lanzhou University Lanzhou Gansu China; ^2^ Department of General Surgery The First Hospital of Lanzhou University Lanzhou Gansu China; ^3^ Department of Epidemiology and Biostatistics, The Seventh Affiliated Hospital Sun Yat‐Sen University Shenzhen Guangdong China; ^4^ Guangdong Provincial Key Laboratory of Gastroenterology, Center for Digestive Disease, the Seventh Affiliated Hospital Sun Yat‐Sen University Shenzhen Guangdong China; ^5^ Chinese Health RIsk MAnagement Collaboration (CHRIMAC) Shenzhen Guangdong China

**Keywords:** bile acids, bile salt hydrolase, gallstone disease, gut microbiota

## Abstract

Gallstone disease is a common global digestive disorder. This study intends to analyze gut microbiota‐gallstone disease interactions, to inform disease mechanism and microbiota‐targeted prevention and treatment strategies. Participants were recruited from health check‐up populations, outpatients, and inpatients. Basic information and biological samples were collected: fecal samples for metagenomic sequencing, and serum samples for bile acid metabolism detection. A total of 62 gallstone patients and 62 healthy controls were enrolled in this study. Compared with the control group, gallstone patients exhibited increased level of bile salt hydrolase (BSH)‐producing bacteria, including the genera *Bacteroides*, *Enterococcus*, *Bifidobacterium*, and the family *Lactobacillaceae*. Further KEGG analysis revealed that the significantly enriched signaling pathways in the gallstone patients were mainly related to bile acid biosynthesis, lipid and bile acid precursor metabolism. Subsequently, we found that in gallstone patients, the levels of hydrophobic bile acids, (e.g., lithocholic acid, LCA), was increased, while the levels of hydrophilic bile acids taurolithocholic acid (TLCA) were decreased. In the correlation analysis between differential bile acids and differential bacterial species, *Bacteroides intestinalis* was positively correlated with LCA, while *Bacteroides fragilis* was negatively correlated with TLCA. These results further confirm the role of BSH‐active bacteria in bile acid dysregulation. This study proposes the “intestinal microbiota imbalance—bile acid metabolic disorder—gallbladder stone formation” axis, and confirms that gallstone patients exhibit intestinal dysbiosis, which leads to bile acid dysregulation. Furthermore, the accumulation of hydrophobic bile acids is identified as a key factor in gallbladder stone formation.

## Graphical abstract

Gallstone patients exhibit intestinal dysbiosis, which leads to bile acid dysregulation. Gallstone patients exhibited increased levels of bacteria with bile salt hydrolase (BSH) activity. In gallstone patients, the levels of hydrophobic bile acids (e.g., lithocholic acid) were increased, while the levels of hydrophilic bile acids (e.g., taurolithocholic acid) were decreased.

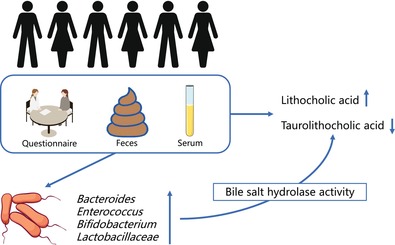

## 1 Background

Gallbladder stones, as one of the most common digestive system diseases worldwide, epidemiological data shows that the global prevalence rate among adults has reached 20%, and about 80% of patients have no obvious clinical symptoms, but with the progression of the disease, about 10%–15% of asymptomatic patients will develop complications such as biliary colic and cholecystitis within 5 years [1, 2]. Surgical removal of the gallbladder becomes the main treatment method, and cholecystectomy has become one of the most common operations performed worldwide [3, 4]. As the primary cause of acute cholecystitis, gallbladder stone‐related acute abdomen accounts for 20%–30% of emergency cases in general surgery, which not only seriously threatens patients' life and health but also brings a heavy burden to the social medical system [5].

In recent years, studies on the association between gut microbiota and metabolic diseases have revealed its key role in the occurrence and development of gallbladder stones. Previous studies have confirmed that gut microbiota can participate in the stone formation process through multiple pathways; it involves mechanisms such as bile acid metabolism, cholesterol absorption balance, regulation of gallbladder contraction function, and activation of local inflammatory responses [6, 7, 8]. Among these, the microbiota‐dependent regulation of bile acid metabolism is considered the most valuable pathway. Primary bile acids cholic acid (CA) and chenodeoxycholic acid (CDCA) synthesized by the liver are mainly secreted into the intestine through the biliary tract after conjugation with taurine or glycine, and approximately 95% is reabsorbed through active transport in the terminal ileum. In the colon, conjugated bile acids are hydrolyzed by gut microbiota‐derived bile salt hydrolase (BSH) before being converted into secondary bile acids, and these free primary bile acids and secondary bile acids are then reabsorbed through the enterohepatic circulation [9, 10]. When the gut microbiota is dysregulated, it can accelerate the decomposition of cholesterol‐solubilizing bile acids such as CDCA, and the increased proportion of secondary bile acids significantly reduces the solubility of cholesterol in bile [11]. This dual effect causes cholesterol to easily precipitate as crystals, which gradually form stones.

Although current studies have focused on the association between gut microbiota and gallbladder stones, analysis of clinical samples remains insufficient. In particular, the corresponding relationship between the metabolic profiles of different microbiota and changes in bile acid components has not been clarified. This study intends to conduct an in‐depth exploration of the association between the structural characteristics of gut microbiota and gallbladder stone formation through the analysis of clinical samples, so as to provide a basis for elucidating the disease mechanism and developing targeted microbiota‐based prevention and treatment strategies.

## 2 Methods

### 2.1 Study Settings and Participant Selection

This study collected participants from the health examination center, outpatients, and inpatients who visited the First Hospital of Lanzhou University, The First Affiliated Hospital, Sun Yat‐sen University, and The Seventh Affiliated Hospital, Sun Yat‐sen University from January 2022 to July 2024. The inclusion criteria for gallstone patients were as follows: aged ≥ 18 years; diagnosed with gallstone; agreed and signed the informed consent form. The exclusion criteria were: pregnant; having other gastrointestinal diseases; having taken antibiotics, probiotics, or prebiotics within 3 months; having uncontrolled chronic diseases; having tumors; having undergone digestive system surgery; and being postorgan transplantation. In the control group, participants had no gallstones, and the rest of the criteria were the same as the above. The included participants will undergo questionnaire surveys and physical examinations, and their clinical information, blood, and fecal samples will be collected. Ethical approval for the study was obtained from the Seventh Affiliated Hospital of Sun Yat‐sen University, with the approval number KY202203501.

### 2.2 Anthropometric and Laboratory Measurement

Demographic characteristics such as age and gender, past medical history, medication history, and lifestyles such as smoking and drinking habits were collected. Physical activity level was calculated based on daily self‐reported exercise duration at three intensity levels (low, moderate, and high). The total weekly metabolic equivalent (MET)‐minutes was computed using standard coefficients (3.3, 4.0, and 8.0 METs, respectively) according to the International Physical Activity Questionnaire (IPAQ) scoring guidelines [12]. Blood and fecal sample collection was performed by professionally trained nurses in the fasting state at the time of enrollment. The collected serum and a portion taken from the final part of fresh feces were all stored frozen at −80°C.

### 2.3 Human Fecal Bacterial Genomic DNA Extraction

DNA extraction from fecal samples was performed using the GNOME DNA isolation kit (MP Biomedicals) according to the manufacturer's instructions. The extracted DNA samples were resuspended in Elution Buffer, and their quality was evaluated by agarose gel electrophoresis and fluorescent dye method. The extracted DNA was stored at −20°C.

### 2.4 Metagenomic Sequencing

The construction of DNA libraries was completed through five key steps: fragmentation, end repair and A‐tail addition, adapter ligation, PCR amplification, and single‐strand circularization. DNA sequencing was supported by BGI Genomics, with gene sequencing analysis performed using the BGISEQ/MGISEQ platform at a sequencing depth of 10 gigabases (Gb) of clean data [13]. Raw data were filtered by removing reads containing adapter sequence contamination and those with N base counts ≥ 3. Meanwhile, quality trimming was performed on the 3′ end of the sequences, retaining bases with an average quality value ≥ 20. Bowtie2 (v2.3.4.3) was used to align clean data with the human reference genome to remove host‐derived DNA sequences, retaining genomic data related to intestinal microorganisms.

### 2.5 Microbial Community Function Annotation

After preprocessing of high‐quality sequencing reads, Megahit v1.2.9 was used for assembly with the “meta‐sensitive” parameter, and sequences shorter than 500 bp were removed. Prodigal v2.6.3 was used for gene prediction in the metagenomic mode (−p meta), and CD‐HIT was used to construct a nonredundant microbial gene set, with the sequence identity threshold set to 0.95 and the minimum coverage threshold set to 0.9. Gene abundance calculation was performed using Salmon software, expressed as normalized TPM values [14]. Functional annotation was predicted using EggNOG mapper v.2.0.1, and relevant pathway and enzyme information was assigned based on the KEGG database.

### 2.6 Bile Acid Detection

One hundred microliters of serum samples were mixed with 400 μL of methanol/acetonitrile solution (1:1). After thorough vortexing for 1 min, the mixture was allowed to stand at −20°C for 1 h, then centrifuged at 12000 rpm at 4°C. Six hundred microliters of the supernatant were collected. After drying, 100 μL of acetonitrile aqueous solution (1:1) was added, followed by vortexing for 30 s to mix well. After that, the samples were centrifuged again at 12000 rpm at 4°C for 15 min, and the supernatant was collected for detection.

Mixed standard solutions of bile acid metabolites with different concentrations (5 ng/mL, 25 ng/mL, 250 ng/mL, 500 ng/mL, 1000 ng/mL, 2000 ng/mL, and 4000 ng/mL) were prepared. Peak area data of each concentration standard were obtained, and a standard curve was plotted with peak area as the ordinate and concentration as the abscissa. Detection was performed using the SCIEX Triple Quad 5500 LC–MS/MS system, and the ion pair information was sourced from AB SCIEX (USA).

### 2.7 Statistical Analysis

For continuous variables, the Mann–Whitney *U* test was used for nonnormal distribution data, and the Student's *t*‐test was applied for normal distribution data to conduct difference analysis; for categorical variables, the chi‐square test was used to compare differences. The arithmetic rank sum test was used to compare differences in microbial α‐diversity indices. β‐diversity was evaluated by Principal Coordinates Analysis (PCoA) based on Bray‐Curtis distance. Statistical significance of group differences was tested using permutational multivariate analysis of variance (PERMANOVA) with the adonis2 function from the “vegan” package in R software (v4.1.0). We calculated the relative abundance of each genus in every sample. For each group, the median relative abundance was then calculated for each genus, and these values were used to construct a stacked bar plot. Linear discriminant analysis Effect Size (LEfSe) was used to identify species with significant abundance differences between groups, with a threshold set at Kruskal‐Wallis test *p* < 0.05, Wilcoxon rank‐sum test *p* < 0.05, and LDA > 2.5 [15] Differences in KEGG pathways were assessed using a two‐sided Student's *t*‐test, with a significance threshold of *p* < 0.05. The correlation between differential bile acids and differential microbiota was analyzed by the Spearman method, and heatmaps were generated using the “pheatmap” package. Statistical analysis of the data was performed using R software, for all microbiome and bile acid analyses, the false discovery rate (FDR) was controlled using the Benjamini‐Hochberg procedure. Due to the exploratory nature of this study and the limited sample size, a less conservative FDR threshold of 0.20 was adopted. Both the unadjusted *p*‐value < 0.05 and FDR < 0.20 were deemed statistically significant.

## 3 Results

### 3.1 Basic Characteristics of Participants

A total of 62 patients with gallstone disease (GD) and 62 health control (NC) were included in this study. The basic characteristics of the two groups are shown in Table 1. The average age of the population was 55.77 years. More than 77.4% of the population had an education level of junior college, undergraduate or below. There were no significant differences between the two groups in terms of age, education level, smoking, alcohol consumption, sleep status, physical exercise, intake of red meat, vegetables and fruits, daily consumption of tea, coffee, sugary beverages and other characteristics.

**TABLE 1:** Basic characteristics of participants in gallstone disease group and control group.

Characteristics	Gallstone (*n* = 62)	Control (*n* = 62)	*p*
Years (Median, IQR)	54.50 (44.25, 62.50)	58.50 (48.00, 68.00)	0.184
Gender, *N* (%)			
Male	23 (37.1)	20 (32.3)	0.706
Female	39 (62.9)	42 (67.7)	
BMI (Median, IQR)	23.86 (21.18, 26.47)	25.12 (23.50, 27.12)	0.184
BMI, *N* (%)			
Normal (< 24)	33 (53.2)	22 (35.5)	0.128
Overweight (24–27.9)	19 (30.6)	28 (45.2)	
Obesity (> 27.9)	10 (16.1)	12 (19.4)	
Education, *N* (%)			
Junior college, undergraduate or below	44 (71.0)	52 (83.9)	0.133
Junior college, undergraduate or above	18 (29.0)	10 (16.1)	
Smoking status, *N* (%)			
Never	51 (82.3)	56 (90.3)	0.064
Current	11 (17.7)	4 (6.5)	
Quit	0 (0.0)	2 (3.2)	
Drinking status, *N* (%)			
Never	52 (83.9)	53 (85.5)	0.781
Current	8 (12.9)	6 (9.7)	
Quit	2 (3.2)	3 (4.8)	
Sleep status, *N* (%)			
Poor quality	31 (50.0)	35 (56.5)	0.589
Good quality	31 (50.0)	27 (43.5)	
Physical exercise^a^, *N* (%)			
High	36 (58.1)	31 (50.0)	0.471
Moderate or low	26 (41.9)	31 (50.0)	
Coffee consumption, *N* (%)			
1–3 times per week or less	58 (93.5)	56 (90.3)	0.742
4–6 times per week or more	4 (6.5)	6 (9.7)	
Tea consumption, *N* (%)			
1–3 times per week or less	36 (58.1)	33 (53.2)	0.718
4–6 times per week or more	26 (41.9)	29 (46.8)	
Sugary beverages consumption, *N* (%)			
1–3 times per week or less	52 (83.9)	54 (87.1)	0.799
4–6 times per week or more	10 (16.1)	8 (12.9)	
Hypertension, *N* (%)	12 (19.4)	15 (24.2)	0.663
Diabetes mellitus, *N* (%)	8 (12.9)	4 (6.5)	0.362
Hyperlipidemia, *N* (%)	5 (8.1)	0 (0.0)	0.068

Abbreviations: IQR, interquartile range; BMI, body mass index.

^a^ Participants were categorized as having low (< 600 metabolic equivalent (MET)·min/week), moderate (600–3000 MET·min/week), or high (≥ 3000 MET·min/week) physical activity.

### 3.2 Gut Microbiota Differences Between Gallstone Disease Patients and Healthy Individuals

**FIGURE 1:**
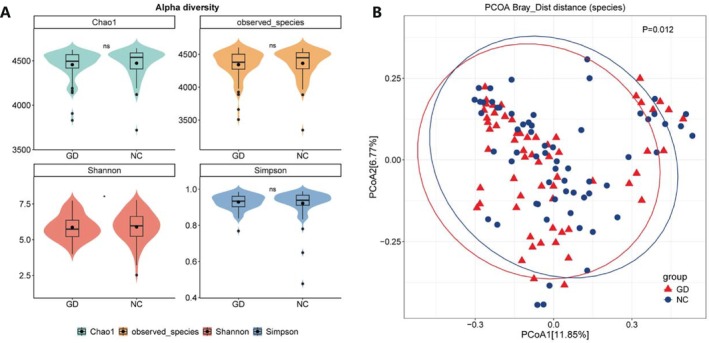
(A) Comparison of gut microbiota diversity between gallstone disease patients (GD) and healthy controls (NC). (A) Gut microbial diversity, Chao1, Observed species, Shannon, and Simpson index. (B) Principal Coordinate Analysis (PCoA) based on Bray‐Curtis distance. The arithmetic rank sum test was used to compare differences in microbial α‐diversity indices. β‐diversity was evaluated by Principal Coordinates Analysis (PCoA) based on Bray‐Curtis distance. Statistical significance of group differences was tested using permutational multivariate analysis of variance (PERMANOVA). *p < 0.05, *n* = 62 (GD) and *n* = 62 (NC).

In the GD group, there were no differences in the Chao1 index, observed species, and Simpson index, while the Shannon index was lower than that in the NC group, suggesting that the gut microbiota richness in gallstone patients is similar with healthy population, but the evenness of species distribution is reduced (Figure 1A). To determine the differences in the bacterial community composition between the GD group and the NC group, we compared the intergroup differences in bacterial communities between the two groups through Principal Coordinate Analysis (PCoA) based on Bray‐Curtis distance, thereby evaluating β‐diversity. The proportion of variance explained by PCoA1 was 11.85%, and that explained by PCoA2 was 6.77% (Figure 1B). The two groups can be clearly distinguished using the distance algorithm, indicating that there were significant differences in the composition of the microbiota between the two groups (*p* = 0.012).

### 3.3 Gut Microbiota Abundance Alterations Between Gallstone Disease Patients and Healthy Individuals

**FIGURE 2:**
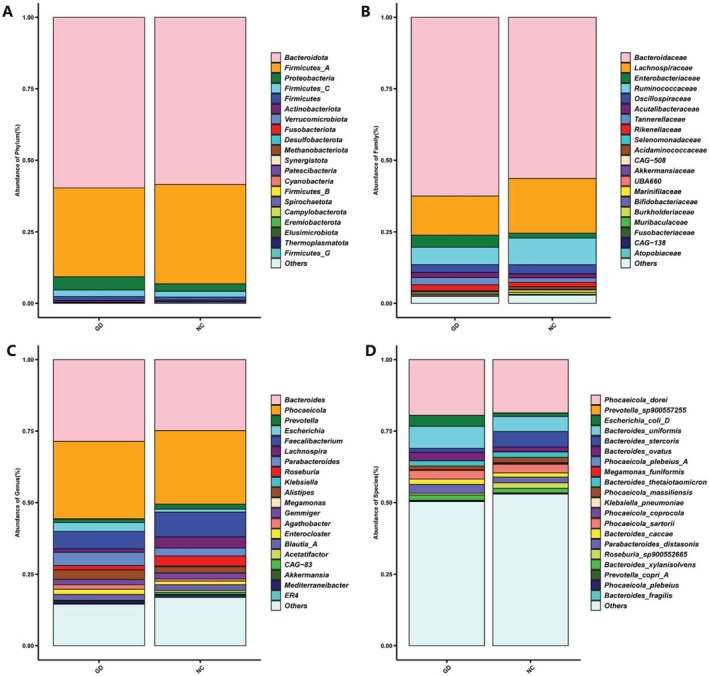
Distribution map of fecal microbiota composition between gallstone disease patients (GD) and healthy controls (NC). Differences in gut microbiota at the phylum (A), family (B), genus (C), species (D) level. Taxonomic stacked bar chart presenting the relative abundances of the top 20 gut bacterial taxa at the respective level in the GD and NC groups. *n* = 62 (GD) and *n* = 62 (NC).

The predominant bacteria at the phylum level in both groups were *Bacteroidota*, *Firmicutes*, and *Proteobacteria*. Compared with the NC group, *Bacteroidota* and *Proteobacteria* exhibited an increasing trend in relative abundance while those of *Firmicutes* showed a decreasing trend in gallbladder stone patients (Figure 2A). At the family level, *Bacteroidaceae* had the highest abundance. Compared with the NC group, a tendency toward reduced relative abundances of *Lachnospiraceae*, *Ruminococcaceae*, and *Oscillospiraceae* was shown in gallstone patients, while those of *Enterobacteriaceae*, *Acutalibacteraceae*, and *Tannerellaceae* had an increasing trend (Figure 2B). At the genus level, the top five most abundant genera in both groups of participants were *Bacteroides*, *Phocaeicola*, *Prevotella*, *Faecalibacterium*, and *Escherichia*. Compared with the NC group, the abundances of *Bacteroides*, *Escherichia*, *Parabacteroides*, and *Alistipes* showed an increasing trend in the GD group, while those of *Prevotella*, *Phocaeicola*, *Faecalibacterium*, and *Lachnospira* showed a decreasing trend (Figure 2C). At the species level, compared with the NC group, the abundances of *Phocaeicola dorei*, *Escherichia coli* , *Bacteroides uniformis* , *Bacteroides ovatus* , *Phocaeicola sartorii*, among others, had an increasing trend in the gallstone patients, while those of *Bacteroides stercoris* , *Phocaeicola massiliensis*, and other species showed a decreasing trend (Figure 2D).

### 3.4 Further Differential Bacterial Composition Analysis of Gallstone Patients

**FIGURE 3:**
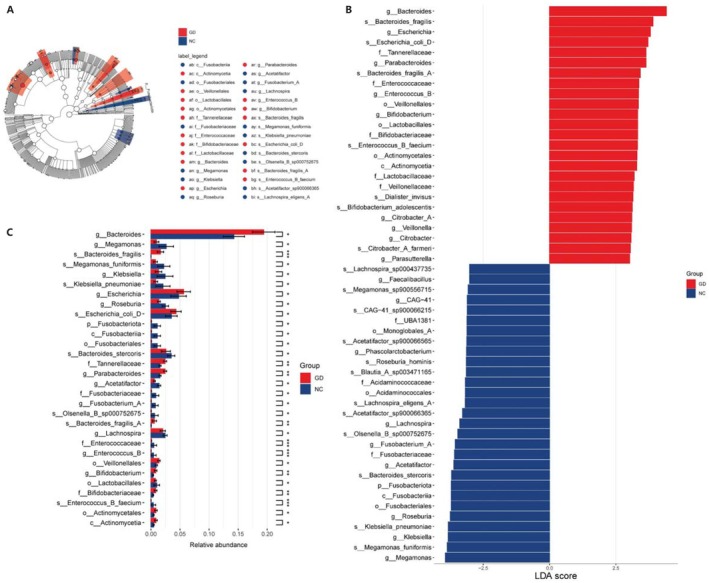
LEfSe analysis of differential microbiota in gallstone disease patients (GD) and healthy controls (NC). (A) Microbial taxonomic hierarchy tree. Red indicates higher microbial abundance in the GD group, and blue indicates higher microbial abundance in the NC group. (B) Microbial features with significant differences between the two groups with linear discriminant analysis (LDA) score > 3.0, LEfSe was used to identify taxa with significant abundance differences between groups, with the Kruskal–Wallis test (*p* < 0.05) for between‐group comparisons, followed by the Wilcoxon rank‐sum test (*p* < 0.05) for consistency. (C) The top 30 differential microbiota between the GD Group and the NC Group. *p < 0.05, **p < 0.01, ***p < 0.001 indicate statistical significance based on the above Kruskal–Wallis and Wilcoxon rank‐sum tests with Benjamini‐Hochberg false discovery rate (FDR) correction (FDR < 0.20). *n* = 62 (GD) and *n* = 62 (NC).

We used LEfSe analysis to compare the differences in gut microbiota abundance between the GD group and the NC group, and LDA score was used to quantify the magnitude of differences in characteristic microbiota between the two groups. The results showed that compared with the control group, the composition of gut microbiota in gallbladder stone patients changed significantly (Figure 3A). Using a threshold of LDA score > 2.5, we found that in the GD group, bacteria including *Roseburia*, *Lachnospira*, *Blautia* were decreased, while bacteria with bile salt hydrolase (BSH) activity such as *Bacteroides*, *Enterococcus*, *Bifidobacterium*, and *Lactobacillaceae* were increased (Figure 3B,C, Table S1). These results indicated that bacteria with BSH activity may play a key role in gallstone formation.

### 3.5 Gut Microbiota Alternations Affect Metabolic Functions

**FIGURE 4:**
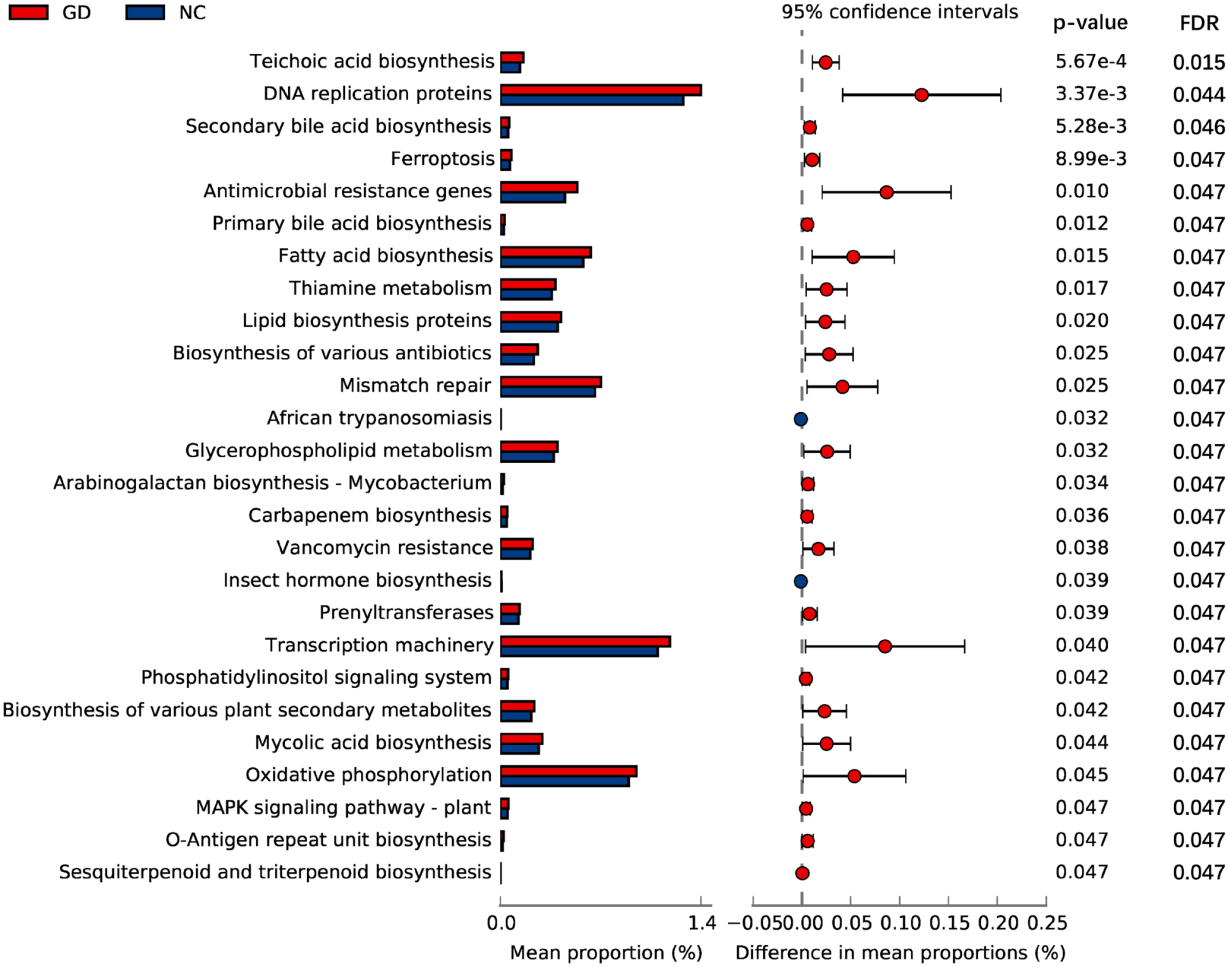
KEGG functional enrichment analysis of differential bacteria between gallstone disease patients (GD) and healthy controls (NC). Differential KEGG pathways were evaluated using a two‐sided Student's *t*‐test, and *p* values were adjusted for multiple testing with the Benjamini‐Hochberg method. Pathways with *p* < 0.05 and false discovery rate FDR < 0.20 were considered statistically significant. *n* = 62 (GD) and *n* = 62 (NC).

KEGG functional enrichment analysis was performed on the differential microbiota, and the statistically significant signaling pathways were mainly enriched in primary and secondary bile acid metabolism, as well as pathways related to lipid and bile acid precursor metabolism, including Fatty acid biosynthesis, and Lipid biosynthesis proteins. Additionally, pathways that may lead to intestinal microbiota dysbiosis were identified, such as antimicrobial resistance genes, along with those related to cell wall synthesis, including Teichoic acid biosynthesis, Mycobacterium, and Mycolic acid biosynthesis (Figure 4). The KEGG results confirmed the presence of intestinal microbiota dysbiosis in the GD group, as well as potential bile acid metabolism disorders.

### 3.6 Characteristics of Serum Bile Acid Change in Gallstone Patients

**FIGURE 5:**
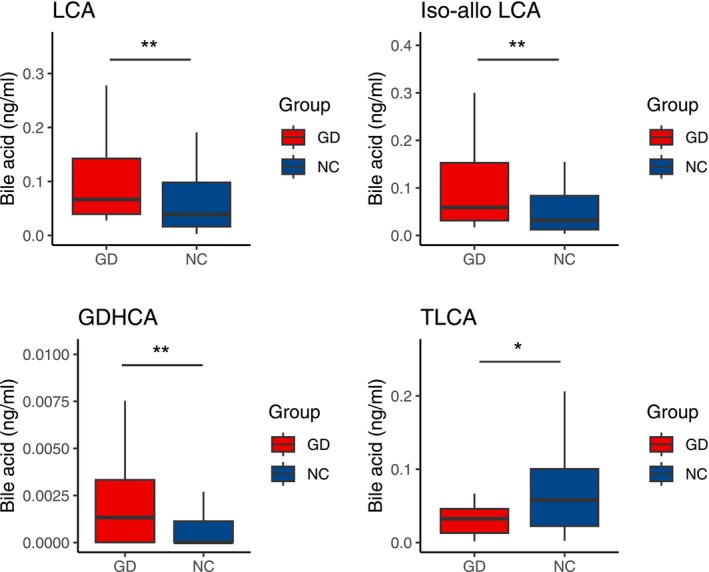
Changes in serum bile acid levels between gallstone disease patients (GD) and healthy controls (NC). Data are presented as median [interquartile range, IQR]. Differences in bile acid levels between groups were assessed using the Wilcoxon rank‐sum test, and *p* values were adjusted for multiple testing using the Benjamini‐Hochberg false discovery rate (FDR) procedure. Statistical significance was defined as a *p* value < 0.05 and an FDR < 0.20. *p < 0.05, **p < 0.01, *n* = 44 (GD) and *n* = 44 (NC).

To further explore the mechanism of the gut microbiota‐bile acid axis in the development of gallbladder stones, we performed targeted metabolomics analysis via LC–MS on qualified serum samples from 44 gallstone patients and 44 healthy controls. The Wilcoxon rank‐sum test was used for statistical analysis of bile acid levels in the two groups. The results showed that compared with the NC group, the GD group had characteristic changes in the bile acid profile, among them, lithocholic acid (LCA), isoallolithocholic acid (Iso‐allo‐LCA), and glycodehydrocholic acid (GDHCA) were increased, while taurolithocholic acid (TLCA) were decreased (Figure 5, Table S2).

### 3.7 Serum Bile Acid Changes Are Associated With Gut Microbiota

**FIGURE 6:**
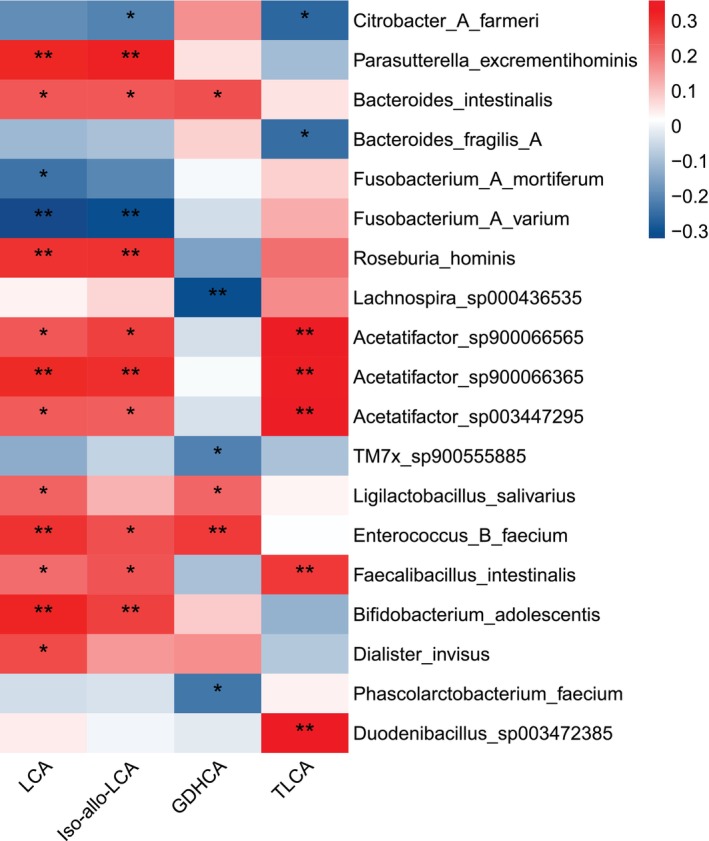
Correlation analysis between differential serum bile acids and differential bacterial species. Red indicates positive correlations and blue indicates negative correlations. The intensity of the color corresponds to the strength of the relationship. *p < 0.05, **p < 0.01, *n* = 44 (GD) and *n* = 44 (NC).

Spearman correlation analysis was performed between the differential bacterial species identified by metagenomics and the differential bile acids. The results showed that *Bacteroides intestinalis* was positively correlated with LCA, while *Bacteroides fragilis* was negatively correlated with TLCA. *Parasutterella*, *Bifidobacterium adolescentis* , and *Roseburia hominis* were positively correlated with LCA and Iso‐allo‐LCA, *Acetatifactor* and *Faecalibacillus intestinalis* were positively correlated with LCA, Iso‐allo‐LCA, and TLCA (Figure 6).

## 4 Discussion

A growing number of studies has focused on the impact of gut microbiota on diseases; however, how gut microbiota influences the formation of gallbladder stones remains inconclusive [16, 17, 18]. Through multidimensional analyses of metagenomic sequencing and bile acid metabolomics, this study systematically reveals the characteristic changes in the structure and function of gut microbiota in patients with gallbladder stones and clarifies its close association with abnormal bile acid components. Our results provide a new perspective for understanding the role of the gut microbiota‐bile acid axis in the pathogenesis of gallbladder stones.

In this study, we found that patients with gallbladder stones had gut microbiota dysbiosis. The decreased α‐diversity Shannon index in gallstone patients indicates that impaired uniformity of the gut microbiota, while the significant difference in β‐diversity confirms the overall structural changes of the microbiota under disease conditions. At the phylum level, gallbladder stone patients show a trend of increased abundances of *Bacteroidota* and *Proteobacteria* and decreased abundance of *Firmicutes*, which represents the imbalance of gut microbiota structure in these patients. In previous studies, an increase or decrease in the *Firmicutes*/*Bacteroidota* (F/B) ratio has been regarded as gut microbial dysbiosis [19, 20, 21]. A decreased F/B ratio is associated with inflammatory bowel disease, depression, Alzheimer's disease, etc. [19, 21, 22, 23, 24]. In addition, changes in gut microbiota structure may modify the intestinal metabolic microenvironment and may also impair the functional robustness of the gut microbiota, which is one of the conditions leading to metabolic disorders of the gut microbiota [25, 26, 27].

In the GD group, analysis at the family level further revealed that the abundance of *Enterobacteriaceae*, which is associated with inflammatory responses, was significantly increased [28]. At the genus level, there were reduced abundances of *Faecalibacterium*, which is an important anti‐inflammatory genus; its decreased abundance may damage the intestinal barrier, promote endotoxin translocation and chronic inflammation, which is likely closely related to the pathophysiological process of gallbladder stone formation [29, 30, 31]. Moreover, the decrease in its abundance is considered a risk factor for biliary system diseases [32].

The results of KEGG functional enrichment indicated the enrichment of antimicrobial resistance genes and cell wall synthesis associated pathways, reflecting the adaptive survival strategies of the microbiota under pathological conditions; it may further alter the imbalance of the microbiota structure [33, 34]. In addition, differential functions were not only enriched in primary and secondary bile acid synthesis but also in the activation of pathways related to lipid and bile acid precursor metabolism (e.g., fatty acid biosynthesis, lipid biosynthesis proteins), and these results are similar to those of previous studies [35, 36, 37]. This suggests that gallstone patients may have bile acid disorders, in which the gut microbiota might provide substrates for the abnormal accumulation of the bile acid pool by enhancing cholesterol synthesis and conversion capabilities.

The abundances of BSH‐positive bacteria such as *Bacteroides*, *Enterococcus*, *Bifidobacterium*, and *Lactobacillaceae* were increased in GD group. BSH can hydrolyze conjugated bile acids and directly regulate the conversion of primary bile acids to secondary bile acids [38]. Their excessive proliferation disrupts the balance of the enterohepatic circulation of bile acids: on the one hand, it accelerates the decomposition of cholesterol‐solubilizing bile acids (e.g., CDCA), Previous studies have shown that there is a negative correlation between BSH and CDCA [11, 39]. On the other hand, it promotes the production of hydrophobic secondary bile acids (e.g., LCA), thereby reducing the solubility of cholesterol in bile [40, 41].

In this study, we found that there were characteristic changes in the bile acid profile in the GD group. Specifically, hydrophobic bile acids LCA and Iso‐allo‐LCA were increased, while protective bile acids taurolithocholic acid (TLCA) were decreased [42, 43]. The hydrophilicity of secondary bile acids is related to the number of hydroxyl groups. LCA is the most hydrophobic bile acid, containing only one hydroxyl group, followed by DCA, which has two hydroxyl groups, and UDCA is used in the treatment of gallstones due to its high hydrophilicity [44]. In our study, LCA was upregulated in the GD group, while no changes in DCA or UDCA were observed. The strong hydrophobicity of LCA makes it difficult to form stable soluble micelles with cholesterol and phospholipids effectively, which significantly reduces the solubility of cholesterol in bile, leading to gradual supersaturation of cholesterol and forming gallstones [45]. Moreover, the accumulation of LCA can induce inflammation of the gallbladder mucosa, impair gallbladder contraction function, and further exacerbate cholestasis and stone formation [46].

In the liver or intestine, free LCA can combine with taurine via an amide bond to form conjugated TLCA, which increases water solubility to facilitate secretion or reabsorption [47]. Previous studies have indicated that 98.5% of *Bacteroides* strains carry the *bsh* gene. Among them, the BSH activity of *Bacteroides fragilis* is significantly higher than that of other *Bacteroides* species; besides, *Enterococcus faecalis* also exhibits BSH enzymatic activity [48]. In the intestine, BSH can specifically break this amide bond, hydrolyzing TLCA into free LCA and taurine [49]. Free LCA can be reabsorbed back to the liver through the enterohepatic circulation or further metabolized and excreted from the body. BSH plays a central role in the enterohepatic circulation and intestinal metabolism of bile acids. In the correlation analysis between differential bile acids and differential bacterial species, *Bacteroides intestinalis* and *Enterococcus faecalis* were positively correlated with LCA, while *Bacteroides fragilis* was negatively correlated with TLCA; it is because the BSH of bacteria tends to decompose TLCA, which leads to an increase in the release of LCA [48, 49]. Such species‐specific BSH substrate preference results in an imbalance in the LCA/TLCA ratio.

In addition, short‐chain fatty acid (SCFA)‐producing bacteria such as *Roseburia*, *Lachnospira*, and *Blautia_A* were significantly reduced; it may lead to a decrease in the protective effect of SCFAs against gallstones [50]. Previous studies have indicated that the abundance of *Roseburia* is downregulated in individuals with gallstones, and the abundance of *Roseburia* is restored after bile acid treatment [51, 52]. Butyrate produced by *Lachnospira* also has a certain protective potential [53, 54]. As for *Blautia_A*, it exhibits activities in maintaining intestinal homeostasis, such as preventing inflammation and promoting the production of SCFAs, and possesses potential probiotic properties [55]. The reduction in these bacteria can lead to impaired intestinal barrier function, indirectly promoting the formation of gallbladder stones [56, 57, 58].

In summary, this study proposes a potential mechanism of “intestinal microbiota imbalance—bile acid metabolic disorder—gallbladder stone formation”: the enrichment of dominant bacterial genera substrates by activating lipid synthesis pathways; meanwhile, BSH secreted by bacteria alters the direction of bile acid transformation, leading to the accumulation of hydrophobicity bile acids such as LCA. On the other hand, the reduction of functional bacterial families under *Firmicutes* impairs the intestinal metabolic buffering capacity and barrier function. These factors collectively promote the imbalance of bile components and the formation of gallstones.

The limitations of this study include a small sample size, and large‐sample, multicenter studies will be conducted in the future. Additionally, there is a lack of verification through animal experiments involving fecal microbiota transplantation or genus‐specific intervention, and the specific changes in the activity of bile acid metabolizing enzymes have not been detected. Future research could combine metatranscriptomics and metabolomics to further dissect the molecular mechanisms underlying the microbiota‐bile acid interaction, with a focus on the role of BSH in gallbladder stone formation.

## Author Contributions

C.H., W.X., and J.Z. were involved in study design, data collecting, paper conceptualization, data analysis, and paper writing. R.Z., Q.H., and B.X. were involved in data collecting and data analysis. L.G., H.M., and L.T. were involved in data collecting and quality control. P.Y. and Y.L. were involved in data collecting and project administration. J.Y, Y.M., and W.M. were involved in study conceptualization and paper editing. W.M. was in charge of the study, and involved in study conceptualization, paper conceptualization, data analysis, project administration and supervision and paper editing. All authors contributed to the interpretation of the data and approved the final version for submission.

## Funding

This work was supported by the National Natural Science Foundation of China (82204123 and 82473707), Funding of Shenzhen Clinical Research Center for Gastroenterology (Gastrointestinal Surgery) (LCYSSQ20220823091203008), and National Key Research and Development Program of China (2022YFC2407405).

## Conflicts of Interest

The authors declare no conflicts of interest.

## Supporting information


**Table S1:** Differential bacteria at the genus and species levels with LDA score > 2.5.

**Table S2:** Changes in serum bile acid levels between gallstone disease patients (GD) and healthy controls (NC).

## Data Availability

The data that support the findings of this study are available on request from the corresponding author. The data are not publicly available due to privacy, legal, or ethical restrictions.
