# Supplementary material for: Gut Microbiome Dysbiosis Promotes Gallstone Formation via Bile Acid Metabolic Disorder: A Multiomics Study
Source: FASEB J. 2026 Mar 9;40(6):e71656. doi: 10.1096/fj.202503254RRRRRR (PMC12968746; doi:10.1096/fj.202503254RRRRRR)
Supplement: Supplementary file 1 — Table S1: Differential bacteria at the genus and species levels with LDA score > 2.5. [file FSB2-40-e71656-s002.docx]

T**able S1. Differential bacteria at the genus and species levels with LDA score > 2.5**

| Genus | Species | Group | LDA score | *p* value | FDR |
| --- | --- | --- | --- | --- | --- |
| Phascolarctobacterium | Phascolarctobacterium_faecium | Control | 2.992 | 0.046 | 0.046 |
| RC9 | RC9_sp000433355 | Gallstone | 2.958 | 0.001 | 0.003 |
| Enterocloster | Enterocloster_clostridioformis | Gallstone | 2.944 | 0.016 | 0.027 |
| Ligilactobacillus | - | Gallstone | 2.939 | 0.022 | 0.033 |
| Mediterraneibacter | Mediterraneibacter_faecis | Control | 2.915 | 0.018 | 0.028 |
| Duodenibacillus | - | Control | 2.874 | 1.93E-05 | 1.74E−04 |
| Faecalibacillus | Faecalibacillus_intestinalis | Control | 2.863 | 4.03E−04 | 0.002 |
| Parasutterella | Parasutterella_excrementihominis | Gallstone | 2.838 | 2.94E-05 | 2.12E-04 |
| Coprobacter | - | Gallstone | 2.826 | 0.012 | 0.021 |
| CAG-273 | CAG-273_sp003534295 | Control | 2.825 | 0.002 | 0.007 |
| Bacteroides | Bacteroides_intestinalis | Gallstone | 2.811 | 0.037 | 0.041 |
| Muribaculum | - | Gallstone | 2.811 | 0.005 | 0.010 |
| Faecalibacterium | Faecalibacterium_sp900539945 | Control | 2.794 | 0.044 | 0.045 |
| Blautia | - | Control | 2.785 | 0.038 | 0.041 |
| Ligilactobacillus | Ligilactobacillus_salivarius | Gallstone | 2.765 | 0.007 | 0.014 |
| Roseburia | Roseburia_sp900542495 | Control | 2.745 | 0.026 | 0.035 |
| Fusobacterium_A | Fusobacterium_A_mortiferum | Control | 2.745 | 5.41E-08 | 9.74E-07 |
| TM7x | - | Control | 2.738 | 0.004 | 0.010 |
| Bacteroides | Bacteroides_faecis | Gallstone | 2.738 | 0.014 | 0.024 |
| Escherichia | Escherichia_fergusonii | Gallstone | 2.733 | 0.035 | 0.041 |
| Fusobacterium_A | Fusobacterium_A_varium | Control | 2.730 | 1.22E-06 | 1.46E-05 |
| Anaerotignum | - | Control | 2.714 | 0.030 | 0.037 |
| TM7x | TM7x_sp900555885 | Control | 2.714 | 0.026 | 0.035 |
| UBA9502 | - | Control | 2.683 | 0.003 | 0.008 |
| Negativibacillus | - | Control | 2.683 | 1.75E-04 | 9.00E-04 |
| Duodenibacillus | Duodenibacillus_sp900538905 | Control | 2.670 | 4.21E-05 | 2.53E-04 |
| Acetatifactor | Acetatifactor_sp003447295 | Control | 2.644 | 0.010 | 0.019 |
| Lachnospira | Lachnospira_sp000436535 | Control | 2.640 | 0.004 | 0.010 |
| Escherichia | Escherichia_sp000208585 | Gallstone | 2.636 | 0.025 | 0.035 |
| Lachnospira | Lachnospira_sp003537285 | Control | 2.610 | 0.033 | 0.039 |
| CAG-302 | CAG-302_sp001916775 | Control | 2.609 | 0.011 | 0.021 |
| Bifidobacterium | Bifidobacterium_pseudocatenulatum | Gallstone | 2.569 | 0.004 | 0.010 |
| CAG-279 | CAG-279_sp000437795 | Gallstone | 2.552 | 0.043 | 0.045 |
| Duodenibacillus | Duodenibacillus_sp003472385 | Control | 2.539 | 8.82E-09 | 3.18E-07 |
| Blautia | Blautia_hansenii | Control | 2.521 | 0.001 | 0.002 |
| Streptococcus | Streptococcus_salivarius | Control | 2.515 | 0.029 | 0.037 |

62 patients with gallstone disease and 62 health control were included. LEfSe was used to identify taxa with significant abundance differences between groups, with thresholds set at Kruskal-Wallis test *p*< 0.05 and Wilcoxon rank-sum test *p*< 0.05. The Benjamini-Hochberg procedure was applied to control the false discovery rate (FDR), and FDR < 0.20 was considered statistically significant.
